# Repurposing Lovastatin Cytotoxicity against the Tongue Carcinoma HSC3 Cell Line Using a Eucalyptus Oil-Based Nanoemulgel Carrier

**DOI:** 10.3390/gels8030176

**Published:** 2022-03-12

**Authors:** Waleed Y. Rizg, Khaled M. Hosny, Samar S. Mahmoud, Ahmed K. Kammoun, Abdulmohsin J. Alamoudi, Hossam H. Tayeb, Haitham A. Bukhary, Moutaz Y. Badr, Samar S. A. Murshid, Eman Alfayez, Sarah A. Ali, Rayan Y. Mushtaq, Walaa A. Abualsunun

**Affiliations:** 1 Department of Pharmaceutics, Faculty of Pharmacy, King Abdulaziz University, Jeddah 21589, Saudi Arabia; kmhomar@kau.edu.sa (K.M.H.); wabuassonon@kau.edu.sa (W.A.A.); 2 Center of Excellence for Drug Research and Pharmaceutical Industries, King Abdulaziz University, Jeddah 21589, Saudi Arabia; 3 Cairo Laboratories for Clinical Pathology, Department of Clinical Biochemistry, Cairo 32194, Egypt; samar_elgebaly@yahoo.com; 4 Department of Pharmaceutical Chemistry, Faculty of Pharmacy, King Abdulaziz University, Jeddah 21589, Saudi Arabia; akammoun@hotmail.com; 5 Department of Pharmacology and Toxicology, Faculty of Pharmacy, King Abdulaziz University, Jeddah 21589, Saudi Arabia; ajmalamoudi@kau.edu.sa; 6 Department of Medical Laboratory Technology, Faculty of Applied Medical Sciences, King Abdulaziz University, Jeddah 21589, Saudi Arabia; hhtayeb@kau.edu.sa; 7 Center of Innovation in Personalized Medicine (CIPM), Nanomedicine Unit, King Abdulaziz University, Jeddah 21589, Saudi Arabia; 8 Department of Pharmaceutics, College of Pharmacy, Umm Al-Qura University, Makkah 24381, Saudi Arabia; habukhary@uqu.edu.sa (H.A.B.); mybadr@uqu.edu.sa (M.Y.B.); 9 Department of Natural Products and Alternative Medicine, Faculty of Pharmacy, King Abdulaziz University, Jeddah 21589, Saudi Arabia; samurshid@kau.edu.sa; 10 Department of Oral Biology, Faculty of Dentistry, King Abdulaziz University, Jeddah 21589, Saudi Arabia; ealfayez@kau.edu.sa; 11 Department of Oral Diagnostic Sciences, Faculty of Dentistry, King Abdulaziz University, Jeddah 21589, Saudi Arabia; samali@kau.edu.sa; 12 Department of Pharmaceutics, College of Clinical Pharmacy, Imam Abdulrahman Bin Faisal University, Dammam 31441, Saudi Arabia; rymushtaq@iau.edu.sa

**Keywords:** statins, essential oils, tongue cancer, experimental design, nanosized delivery systems

## Abstract

Tongue cancer is one of the most common carcinomas of the head and neck region. The antitumor activities of statins, including lovastatin (LV), and the essential oil of eucalyptus (Eu oil), have been adequately reported. The aim of this study was to develop a nanoemulgel containing LV combined with Eu oil that could then be made into a nanoemulsion and assessed to determine its cytotoxicity against the cell line human chondrosarcoma-3 (HSC3) of carcinoma of the tongue. An I-optimal coordinate-exchange quadratic mixture design was adopted to optimize the investigated nanoemulsions. The droplet size and stability index of the developed formulations were measured to show characteristics of the nanoemulsions. The optimized LV loaded self-nanoemulsifying drug delivery system (LV-Eu-SNEDDS) was loaded into the gelling agent Carbopol 934 to develop the nanoemulgel and evaluated for its rheological properties. The cytotoxic efficiency of the optimized LV-Eu-SNEDDS loaded nanoemulgel was tested for cell viability, and the caspase-3 enzyme test was used against the HSC3 cell line of squamous carcinoma of the tongue. The optimized nanoemulsion had a droplet size of 85 nm and a stability index of 93%. The manufactured nanoemulgel loaded with the optimum LV-Eu-SNEDDS exhibited pseudoplastic flow with thixotropic behavior. The developed optimum LV-Eu-SNEDDS-loaded nanoemulgel had the best half-maximal inhibitory concentration (IC_50_) and caspase-3 enzyme values of the formulations developed for this study, and these features improved the ability of the nanoemulsion-loaded gel to deliver the drug to the investigated target cells. In addition, the in vitro cell viability studies revealed the synergistic effect between LV and Eu oil in the treatment of tongue cancer. These findings illustrated that the LV-Eu-SNEDDS-loaded gel formulation could be beneficial in the local treatment of tongue cancer.

## 1. Introduction

With the exception of skin cancers other than melanoma, tumors of the oral cavity are the most common cancers in the neck and head region. The tongue, lips, and internal surface of the jaw are the most frequent sites of tumors in the oral cavity; they usually result from the unrestrained division of squamous cells on the tongue’s surface [1,2]. Although many important and advanced steps have been taken in the treatment and diagnosis of oral carcinoma in recent decades [3], the prognosis for advanced-stage tongue cancer is only 50% in patients who survive for 5 years [1]. The paramount risk factors for developing tongue cancer include alcohol addiction and heavy smoking [4]. Less recognized but important triggers are radiation vulnerability, immunity-related disorders, poor oral hygiene, and hereditary factors [5]. In addition, the human papillomavirus plays a substantial role in tongue cancer development [6].

Three-hydroxy-3-methylglutaryl coenzyme A (HMG-CoA) reductase inhibitors, also known as statins, are anti-hypercholesterolemic agents used very often for lowering the incidences of cerebrovascular and cardiovascular diseases [7]. Researchers have also explored the statins’ antitumoral activity against several carcinomas, such as prostate [8], breast [9], esophageal [10], and ovarian malignancies [11]. Lovastatin (LV), also known as mevinolin or monacolin K, is a naturally occurring compound obtained from plants of the *Dioscorea* species, from rice that has fermented due to *Aspergillus* or *Monascus*, or from the *Pleurotus ostreatus* fungus [12,13]. The anticancer effect of LV was attributed to its activity in inhibiting the proliferation of cells and enhancing apoptosis in various carcinomas, such as cervical [14], liver [15], breast [16], and colon [17] cancers. One of the barriers to the use of LV in convenient dosage forms is the drug’s poor aqueous solubility, which leads to poor oral bioavailability (<5%). New dosage forms are needed that can be used through alternative routes [18]. LV was combined with other chemotherapeutic agents to lower cancer cells’ resistance to drugs, and it appreciably improved the therapeutic effect of such agents [19].

Eucalyptus essential oil (Eu oil) is extracted from the leaves of the eucalyptus tree (Myrtaceae family, which includes more than 300 species). Originally, this oil was used in cosmetics, foods, and beverages [20]. Eu oil was also used as an expectorant, antioxidant, and anti-inflammatory agent for disorders such as rheumatism [21].

Eu oil has been reported to be beneficial in cancer management [22]. It was found that the essential oil of eucalyptus species had good cytotoxic activities against the Hela and J774A.1 cell lines in MTT assays [23]. Such a valuable antitumor effect could be related to its content of terpenes, such as α-pinene and γ-terpinene. The suggested mechanism of the anticancer effect of Eu oil could be its ability to promote cell death through an apoptotic process due to its terpene content [24].

The ideal drug delivery system should deliver its cargo with the best therapeutic effect and minimum side effects [25]. Therefore, drug delivery systems of nanosize could be substitutes for imitative ones [26,27]. Nanoemulsions (NEs) are systems formed of surfactant nanoglobules of 20 to 200 nm [28,29]. NEs have several traits not found in classic emulsions that make them exemplary candidates for drug delivery. Such merits include (1) delivering a drug to a target site with reasonable precision due to a minute droplet size, which can affect a large surface area, (2) protecting active agents from hydrolysis or enzymatic degradation, (3) promoting drug loading, solubility, and pharmacokinetic features, (4) diminishing interpatient and intrapatient variations, and (5) controlling the release of studied drugs [30,31].

Nanoemulgels are quite desirable forms for drug delivery due to the dual merits of a nanosized emulsion in a gel base in a single formulation [32]. The NE of the nanoemulgel offers the advantages listed previously, and the gel base provides thermodynamic stability to the prepared emulsion by enhancing the viscosity of the aqueous phase [33]. Furthermore, nanoemulgels have coveted rheological properties that are desirable for topical delivery to sites such as the skin and oral cavity [34]. Interestingly, these dosage forms could improve drug permeation and diffusion by taking advantage of the combined effect of the nanosize of the emulsion globules and certain penetration enhancers.

Experimental design is a recently employed tool that serves such an aim. Its procedures can lead to a “superior solution” that can be obtained through fewer trials to yield an optimized formulation [35]. Additionally, a statistical design can optimize the formulation composition and process and reveal issues that might arise during a study. It can help in identifying the most valuable independent variables using screening methodologies [36]. The experimental design can reveal a formulation’s performance without the need to actually prepare the formulation, and this can save effort and money [37]. To our knowledge, no previous work has explored the synergistic effect of LV and Eu oil in combination in cancer management. Therefore, the objective of the current investigation was to evaluate the antiproliferative activity of LV and Eu oil when formulated in a nanoemulgel against the tongue carcinoma HSC3 cell line.

## 2. Results and Discussion

### 2.1. Estimation of LV Solubility in Various SNEDDS Components

**Figure 1:**
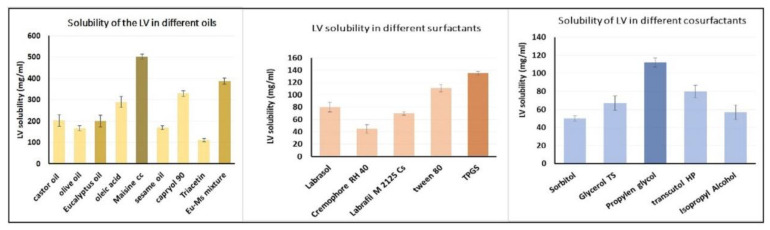
Solubility of LV in different oil phases, surfactants, and co-surfactants.

The maximum solubility of LV was found to be 501 mg/mL in Maisine plus oil, so a mixture of Maisine and Eu oil in a ratio of 1:1 was prepared and the LV solubility in this mixture was determined to be 388 mg/mL. This mixture was used as the oil phase in the formulation of the SNEDDS. The maximum solubility of LV was 135 mg/mL in TPGS (surfactant) and 112 mg/mL in propylene glycol (co-surfactant); therefore, they were considered the surfactant and co-surfactant of choice. The results for LV solubility in different oils, surfactants, and co-surfactants are presented in Figure 1.

### 2.2. Pseudoternary-Phase Diagram for LV in Various Solvent Systems

**Figure 2:**
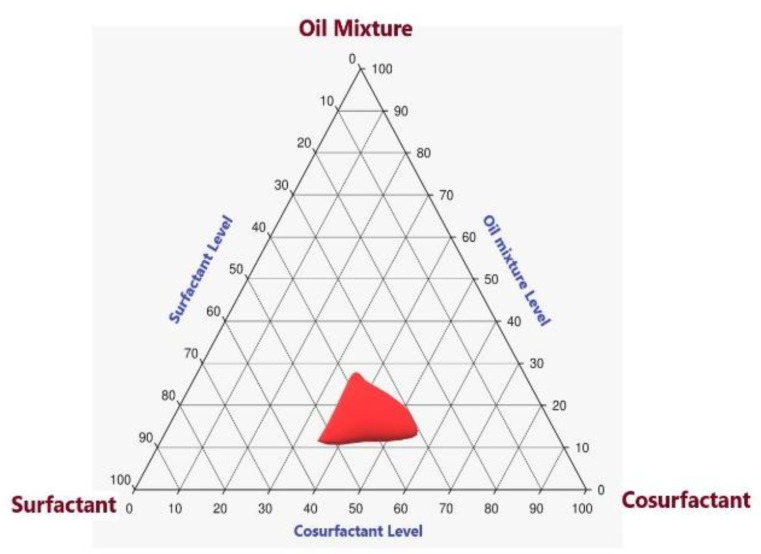
The pseudoternary-phase diagram of Eu oil/Maisine mixture, surfactant (TPGS), and co-surfactant (propylene glycol).

It is noteworthy that the NE region in the pseudoternary-phase diagram (Figure 2) illustrated that the Eu oil/Maisine mixture level could range from 8% to 18%, whereas the TPGS surfactant level ranged from 35% to 50% and the propylene glycol level ranged from 20% to 40%. Therefore, the three components were used within the stated ranges to develop the various LV-Eu-SNEDDS as per the adopted experimental design.

### 2.3. Assessment of LV-Eu-SNEDDS

#### 2.3.1. Visual Inspection of LV-Eu-SNEDDS

Visual examination of the developed LV-Eu-SNEDDS revealed transparent, clear dispersions with no visible clumps. This implied the precision of the employed concentration levels of NE components and the development of physically stable dispersions.

#### 2.3.2. Determination of Droplet Sizes of LV-Eu-SNEDDS

The examined NEs had droplet sizes of between 75 and 170 nm and a polydispersity index of between 0.05 and 0.33. This showed the adequate homogeneity of the fabricated SNEDDS (Table 1).

A special quadratic model of polynomial analysis attained the most significant mean-squared value and surpassed the residual error (*p* < 0.0003), so it was credited with the analysis of the droplet size data. The following experimental design showed the efficiency of the resulting model for evaluating the significant leverage of the Eu oil/Maisine mixture percentage (A), TPGS percentage (B), and propylene glycol percentage (C) on the size of the LV-Eu-SNEDDS droplets. The adjusted R^2^ value (0.9967) of the observed model was in close accordance with its expected R^2^ value (0.8595) (Table 2). The ANOVA analysis of obtained data was used in the following equation:

(1) Droplet size=+136.72A+127.09B+170.27C−207.13AB−302.03AC+32.48BC+1834.80A2BC+137.09AB2C−2744.12ABC2 

**Figure 3:**
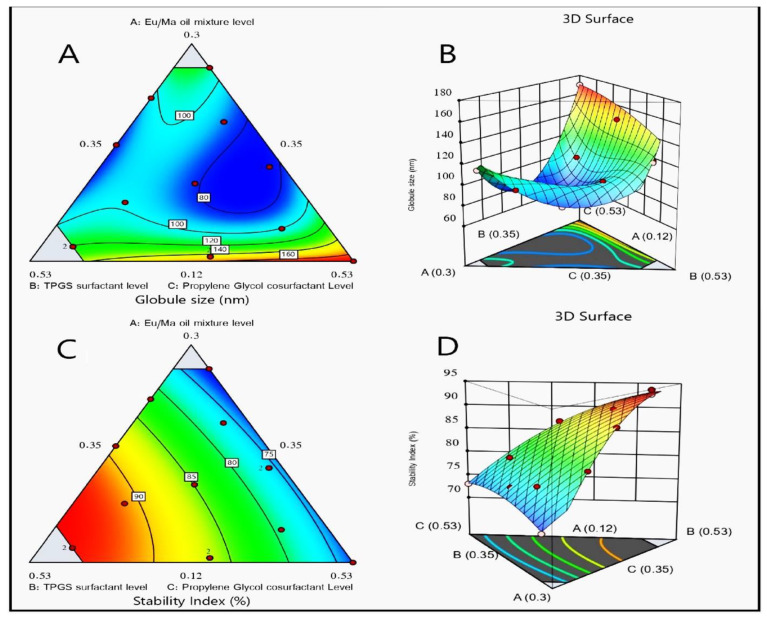
Statistical design plots for the droplet size and stability index of LV-Eu-SNEDDS: (**A**) contour plot for droplet size, (**B**) response surface plot for droplet size, (**C**) contour plot for stability index, and (**D**) response surface plot for stability index.

Figure 3 illustrates the three-dimensional surface and contour plots. They show the effects of the factors on the NE droplet size. This reveals that the NEs had the smallest droplet size when the three independent variables were in their middle levels. This makes sense because the adopted statistical design was a mixture design and the summation of all factors was unity; consequently, increasing one variable would lead to a decrease in the other variables.

#### 2.3.3. Stability Index Assessment

All the tested formulations passed the heat-cool test with no significant signs of instability. The stability index of the NEs is a parameter of paramount importance in assessing their stability. The LV-Eu-SNEDDS exhibited stability index values of between 71% and 94% (Table 1). These values are quite reasonable and make the NE quite stable; this might be ascribed to the minute size of the emulsion droplets, which could improve their physical stability by reducing the expected Ostwald ripening [38].

The quadratic model of polynomial analysis was the proposed model for analyzing the observed results of the second response, as elucidated by the mixture design. The suggested statistical model had an adjusted R^2^ value of 0.9880 and an expected R^2^ value of 0.9761, as seen in Table 2. The ANOVA analysis of gathered data yielded the following equation:

(2) Stability index=+70.19A+93.89B+73.16C+31.10AB+9.52AC+17.24BC 

As seen in Figure 3, which shows the contour and three-dimensional surface plots, factor (B), the TPGS percentage, had the most prominent effect on the stability index. Such a finding could be attributed to the surface-active character of TPGS, which enables it to form a stable film around the droplets of the internal phase, hence promoting their distribution in the continuous phase and minimizing their coalescence and possibility of phase separation [39].

### 2.4. Optimization of LV-Eu-SNEDDS

**Figure 4:**
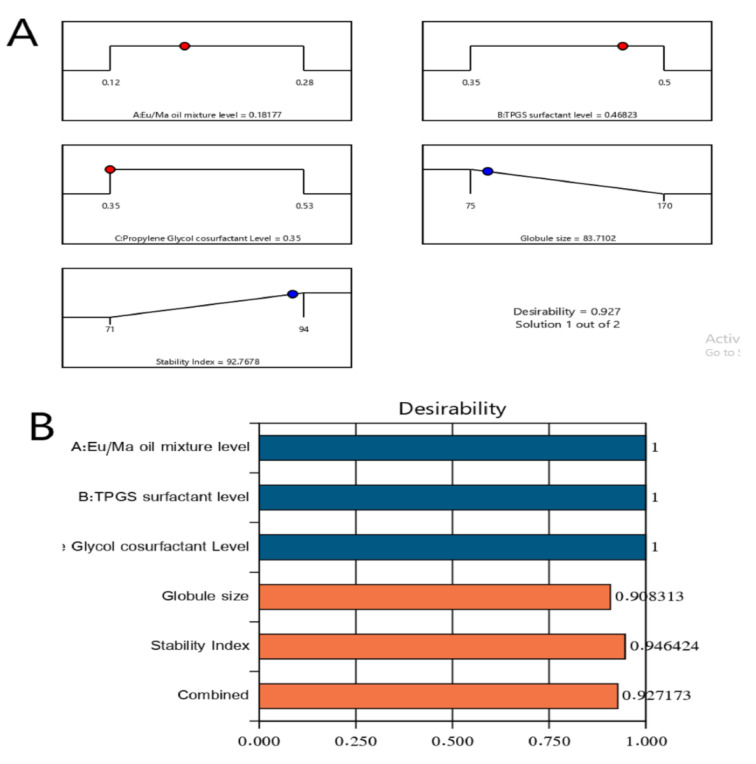
Bar chart and desirability ramp for optimization process. The desirability ramp illustrates the levels of studied factors and expected values for the dependent variables of the optimized LV-Eu-SNEDDS (**A**). The bar chart illustrates the values of desirability for the conjugated responses (**B**).

The ultimate goal of the optimization process of experimental procedures is to determine the most appropriate level of the investigated independent variables to obtain a formulation with the best desired responses [40]. Therefore, Design–Expert software employed the desirability function (D) to optimize the data obtained from the experimental procedures. The software proposed diverse solutions with sundry combinations of the levels of studied independent variables. The desirability plot illustrated a desirability value of 0.927. The engineered model formulation had 0.182% of the Eu oil/Maisine mixture, 0.468% of the TPGS, and 0.350% of the propylene glycol, and it achieved a droplet size of 85 ± 2 nm and a stability index of 93 ± 2%. Additionally, the optimal preparation attained a polydispersity index of 0.371 ± 0.006 and a zeta potential value of 21.6 ± 2.1 mV, indicating good formulation homogeneity and adequate stability. Figure 4 displays the bar chart that explicates the desirability values for the combined responses, along with the desirability ramp that shows the best levels for the studied factors and predicted values of the optimal formulation responses. As seen in Table 3, the divergences detected in the predicted and expected values of the optimized formulation’s responses were found to be insignificant (*p* > 0.05); this boosted the validity and thoroughness of the developed equations. The optimized LV-Eu-SNEDDS was incorporated successfully within the Carbopol base gel, and the results of drug loading of the LV within the prepared optimized gel indicated that the gel was loaded with 20 ± 1.5 mg/mL of LV.

### 2.5. Evaluation of the Optimized LV-Eu-Nanoemulgel

**Figure 5:**
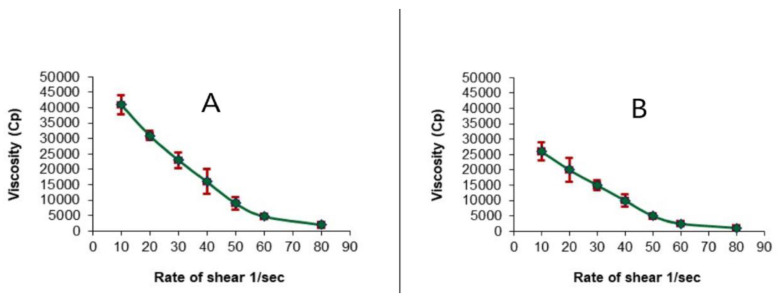
Plots of the shear rate (G) versus the viscosity (η) for (**A**) nanoemulgel loaded with optimized LV-Eu-SNEDDS and (**B**) Carbopol 934 hydrogel (plain). Values are expressed as the mean ± SD (*n* = 3).

The rheological property of a semisolid drug carrier is a very important physical parameter for its application. Figure 5 illustrates the flow curves of the nanoemulgel loaded with the optimized LV-Eu-SNEDDS and Carbopol 934 plain hydrogel. Both possessed good thixotropy, and their viscosity decreased with an increase in the shear rate; one can see that the nanoemulgel exhibited a higher viscosity, apparently owing to its NE content [41]. Thixotropy indicated that the viscosity of the fluid decreased with the increase of shear stress. After the shear stress was removed, the viscosity slowly returned to its former state under isothermal conditions.

**Figure 6:**
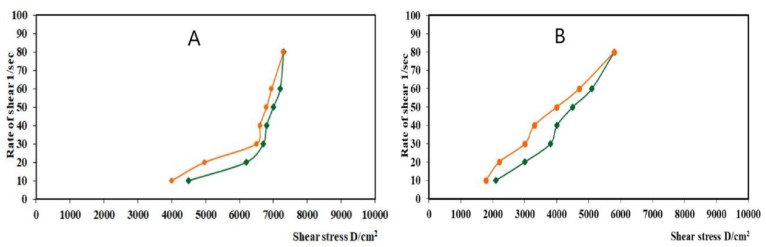
Rheograms of (**A**) nanoemulgel loaded with optimized LV-Eu-SNEDDS and (**B**) Carbopol 934 hydrogel (plain).

Complete rheograms were obtained by plotting the shearing rates as a function of shear stresses (Figure 6). The figure shows a counterclockwise hysteresis curve, or hysteresis loop. Both gel formulations showed pseudoplastic flow with variable thixotropic behavior.

**Figure 7:**
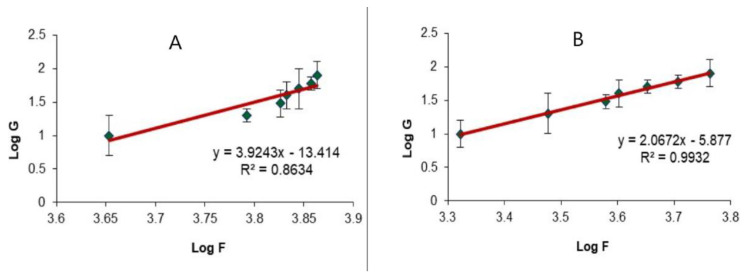
Plots of the logarithm of the shear rate (G) versus the logarithm of the shear stress (F) for (**A**) nanoemulgel loaded with optimized LV-Eu-SNEDDS and (**B**) Carbopol 934 hydrogel (plain). Values are expressed as the mean ± SD (*n* = 3).

The thixotropic behavior of the prepared gel formulations was analyzed using Farrow’s equation, as illustrated in Figure 7. It was found that the nanoemulgel loaded with the optimized LV-Eu-SNEDDS had a better flow behavior and degree of thixotropy compared with the plain gel base.

Thixotropy is important for the topical application of a drug. When a topical preparation is subjected to a shear force, its network structure breaks down, leading to a gradual decrease in viscosity as it is spread on a surface. When the shear force is removed, the viscosity recovers slowly, and this increased viscosity keeps the preparation on the surface to which it has been applied [42].

**Figure 8:**
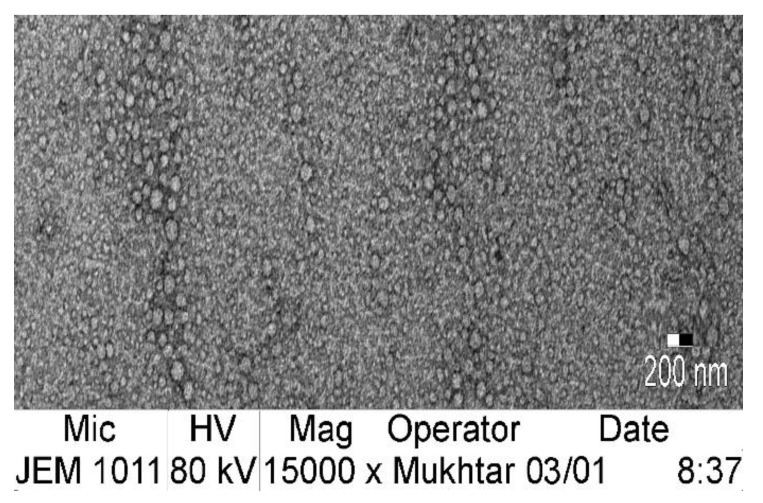
SEM for the optimized nanoemulgel loaded with the optimized LV-Eu-SNEDDS.

The SEM image for the optimized nanoemulgel loaded with the optimized LV-Eu-SNEDDS (Figure 8) indicated that the SNEDDS dispersed within the gel base and the globule size of the dispersed oil globule were less than or nearly equal to 100 nm, and this confirmed the globule size measurement.

### 2.6. In Vitro Release of LV from Different Tested Formulations

**Figure 9:**
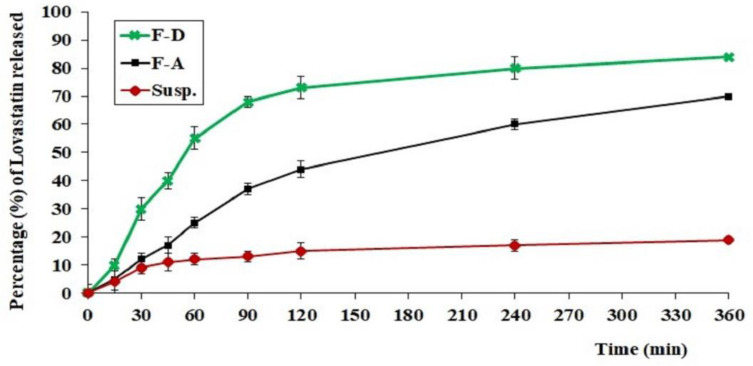
In vitro release profiles of LV from different formulations.

As can be noted in Figure 9, Carbopol gel loaded with the optimized LV-Eu-SNEDDS (A) exhibited homogenous and controlled release of the LV over the duration of the test and the percentage of release of LV reached 70% after 6 h. The release of LV from the tested formulation (D) was rapid, and 86% was released by the end of the test. This was due to the viscosity of the Carbopol gel base, which retarded the rapid initial burst of release of LV in formulation A. In the tested drug suspension, only 20% of the drug dissolved during the test period, and this could be due to the low solubility of LV, whereas, in formulations A and D, the LV entrapped within the SNEDDS was in the nano range, and this provided a large surface area for drug release. Moreover, the drug was present in the solubilized form in the lipid components of the SNEDDS, and this facilitated its dissolution and release.

### 2.7. In Vitro Cell Viability and Caspase-3 Enzyme Assay

The MTT assay was used to assess the in vitro cytotoxic activity of formulations A, B, C, D, and E (previously described in Table 2) against the HSC3 cell line after 72 h of incubation. Figure 8 shows the differences in the percentages of cell viability of the developed formulations; the cell viability was set at 100% for the control group.

**Figure 10:**
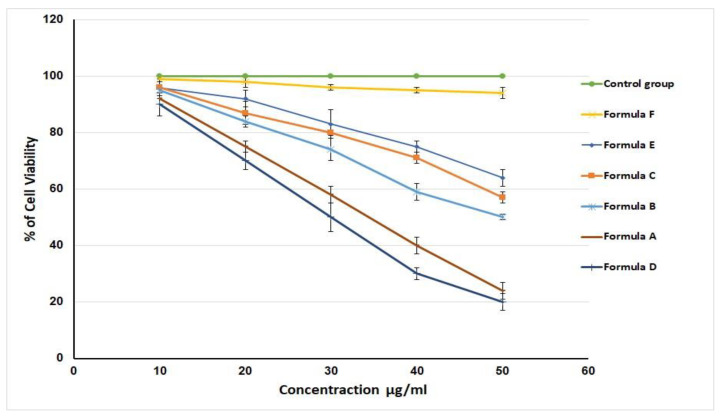
Effect of different LV formulations and 5-fluorouracil (control) on the viability of the HSC3 cell line. The values represent the mean ± SD of three independent experiments (*n* = 9).

Figure 10 affirms that all of the investigated formulations had decreased cell viability in a dose-dependent way. IC_50_ values were 100 ± 1%, 95 ± 2.1%, 64 ± 3.5%, 58 ± 1%, 50 ± 1.5%, 25 ± 3.5%, and 20 ± 4.2% for the control group, F, E, C, B, A, and D formulations, respectively. The LV-Eu-SNEDDS-loaded nanoemulgel (formulation A) obviously had a very low value of IC_50_ compared with formulations B, C, E, and F. This clarifies that formulation A’s substantial cytotoxic activity against HSC3 cells was due to the high affinity of LV for the transporter P-glycoprotein and its consequent high level of cell penetration [43]. Formulation D (i.e., dispersion of LV-Eu-SNEDDS) had an IC_50_ value slightly less than that of formulation A; however, the difference between the IC_50_ values of formulations A and D was found to be insignificant (*p* < 0.05). Such a result reveals the superiority of the developed NE in promoting the permeation of active agents through a cell wall.

The lower IC_50_ values for formulation D (SNEDDS in aqueous dispersion) can be explained by the higher release verified for this formulation when compared with a SNEDDS incorporated in the hydrogel (formulation A). Formulation D was tested against normal HSC3 cells and found to have no cytotoxic effect on the tested cells. Such an outcome clarifies the selectivity of the investigated formulation against cancer cells via apoptosis induction and cell cycle arrest.

**Figure 11:**
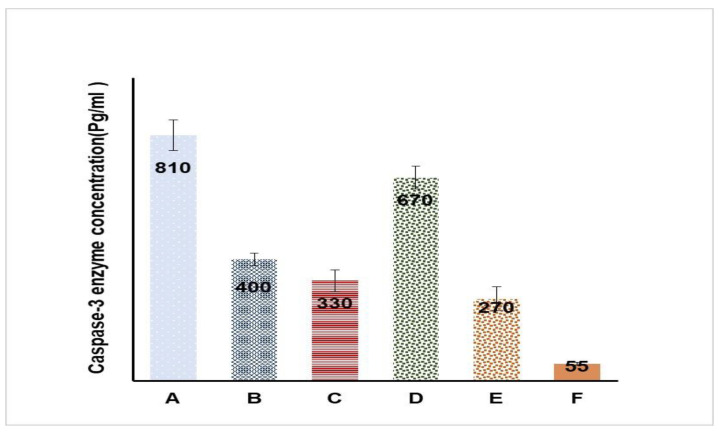
Caspase-3 enzyme concentrations in HSC3 cells treated with different formulations; data are presented as the mean ± SD (*n* = 6). Results were statistically tested using the one-way ANOVA followed by the post-hoc Tukey HSD test.

Figure 11 presents the caspase-3 enzyme levels in cells treated with the studied formulations. It was observed that formulation A had the highest level of the caspase-3 enzyme (810 ± 103 pg/mL) compared with the other formulations (400 ± 26, 330 ± 33, 270 ± 50, and 55 ± 6.4 pg/mL for formulations B, C, E, and F, respectively). Formulation D also had a high concentration of the caspase-3 enzyme (670 ± 65 pg/mL), but the level was lower than that of formulation A. This could be ascribed to the higher viscosity of formulation A, which could have allowed for more intimate contact with cells.

Statins are reported to enhance apoptosis through the activation of the caspase-3 enzyme and the inhibition of geranylgeranyl pyrophosphate (GGPP) biosynthesis, leading to the inhibition of the prenylation of small G-proteins [44]. These findings explain the cytotoxic ability of statins [45]. Additionally, Eu oil exerts a potentially cytotoxic effect owing to its terpene content, which also promotes apoptosis [24]. Such facts explain the high cytotoxic activity of LV-Eu-SNEDDS emulgel formulations against HSC3 cells; it results from the synergistic effect between LV and the Eu oil. Formulation D was tested against a normal HSC3 cell line, and no increase in the capsase-3 enzyme level was observed; this implied the selectivity of the investigated formulation against cancer cells for the previously mentioned reasons.

The data for the capsase-3 enzyme test were tested for normality using the Kolmogorov-Smirnov test of normality. The low D-values and high *p*-values obtained for all formulations suggested that all of the data were not different significantly from data that are normally distributed. The results of the K–S test are summarized in Table 4.

The ANOVA revealed that all formulations exhibited significantly higher caspase-3 enzyme values compared with formulation E (containing plain gel) and had a *p*-value of less than 0.01, so variations between formulations could not be due to chance. The post-hoc Tukey HSD test showed that the caspase-3 enzyme results of all formulations were significantly different from each other except for the comparison between formulations C and B, for which the result was found to be insignificant. Such a result was expected because each of these two formulations contained one component (i.e., LV in B and Eu oil in C), which had an effect on the caspase-3 enzyme values. Results of the post-hoc test are presented in Table 5. A supplemental file containing Tables S1–S6 explains the statistical results more extensively. All formulations exhibited significantly higher caspase-3 enzyme values compared with formulation E (containing plain gel), at a *p*-value of less than 0.01.

## 3. Conclusions

LV was successfully formulated as an NE with adequate characteristics. The most suitable NE regions were assessed using the pseudoternary-phase diagram to obtain the concentrations of Eu oil, TPGS, and propylene glycol. The droplet size of the fabricated NEs ranged between 75 and 170 nm with proper homogeneity. The stability index of the developed formulations was 71% to 94%. The optimal formulation had reasonable stability (93%) and quite a small droplet size (85 nm). Moreover, the optimized LV-Eu-SNEDDS was successfully incorporated into a Carbopol 934 gel base to form a nanoemulgel formulation that had satisfying pseudoplastic flow properties with thixotropic activity. The nanoemulgel loaded with the optimal formulation showed enhanced cytotoxic activity against the HSC3 cell line compared with the other tested formulations, as proved by its very low IC_50_ value and high caspase-3 enzyme level. Thus, this investigation revealed the synergistic cytotoxic effect of LV and Eu oil on cancer cells and the great potential for the use of this combination in nanoemulgels in treating tongue cancer.

## 4. Materials and Methods

### 4.1. Materials

Lovastatin was obtained from Merck KGaA (Darmstadt, Germany). The clove oil, propylene glycol, isopropyl alcohol, and Eu oil were procured from the Tedia Company (Fairfield, OH, USA). Labrasol, Transcutol HP, Maisine CC, Capryol 90, and Labrafil M 2125 CS were generously gifted by Gattefosse (Saint-Priest, France). Sorbitol, glyceryl tristearate, tocopheryl polyethylene glycol succinate (TPGS), Tween 80, triacetin, sesame oil, oleic acid, olive oil, Carbopol 934, castor oil, methanol, and hydroxypropyl methylcellulose (HPMC) were purchased from Sigma-Aldrich (St. Louis, MO, USA). The HSC3 cell lines were a gift from the Department of Pharmacology, King Abdulaziz University, Saudi Arabia. All other solvents, chemicals, and reagents used were of analytical grade.

### 4.2. Estimation of LV Solubility in Various SNEDDS Components

Diversified oils such as oleic acid, castor oil, Eu oil, olive oil, triacetin, Capryol 90, Maisine CC, and sesame oil were used for LV solubility determination. Further, LV solubility was studied in varying surfactants, such as Tween 80, Labrafil M 2125 CS, Cremophor, TPGS, and Labrasol, and in co-surfactants, such as propylene glycol, sorbitol, glyceryl tristearate, isopropyl alcohol, and Transcutol HP. The solubility study was carried out as follows: An excess LV amount was resolved in 3 mL of each tested liquid, and the mixture obtained was carefully blended in a shaking water bath (Model 1031, GFL Corporation, Burgwedel, Germany) at 25 ± 0.5 °C for 72 h. After equilibrium was reached, 1 mL of each mixture was roughly collected and centrifuged (AIC Micro Centrifuge, Boerne, TX, USA) for about 10 min at 1500 rpm. The collected supernatants were diluted with methanol as required. The final concentration of LV was attained using the spectrophotometric method of analysis (Jenway 7315; Bibby Scientific Ltd., Stone, UK) at 238 nm. All measurements were done in triplicate. The data obtained were tested statistically using SPSS software (version 22, Chicago, IL, USA) at a significance level of a *p*-value of less than 0.05.

### 4.3. Pseudoternary-Phase Diagram for LV in Various Solvent Systems

The extent of the LV solubility in the selected liquids (a mixture of Eu oil and Maisine CC; the surfactant TPGS; and the co-surfactant propylene glycol) was the basis for the construction of the LV ternary-phase diagram to find the appropriate ratios for developing SNEDDS. The sum of the three parameters’ concentrations was kept at 100%. Dilution with a water test was done to test the mixture design; the mixture should be able to produce a clear NE after dilution. Different combinations of NE components were used to investigate the NE region in the constructed pseudoternary-phase diagram.

### 4.4. Optimization of LV-Loaded SNEDDS as per Mixture Design

The I-optimal coordinate-exchange quadratic mixture design was used to prepare the LV-loaded SNEDDS using Design-Expert software (version 13.0.7.0, Stat-Ease, Inc., Minneapolis, MN, USA). The design inspected the effect of the three independent variables, namely, the concentrations of the oil mixture (Eu oil/Maisine CC) (A), TPGS (B), and propylene glycol (C). All these factors were used in different ratios, and the total concentration was kept at 100%. The mean droplet size (Y_1_) and stability index (Y_2_) were chosen as the dependent responses. A total of 16 formulations were developed in a random manner (20 mg of LV in each formulation). The variables and their levels selected for each NE formulation are shown in Table 6. The relevance of the studied factors and the determined responses was further investigated with regression equations and statistical analysis strategies applying Design–Expert software. All the formulated dispersions were estimated for the ability to emulsify and the appearance of a formed NE. The formulation with the smallest droplet size and highest stability index was selected as the optimal formulation and consequently used for the preparation of the LV-Eu-nanoemulgel.

### 4.5. Preparation of LV-Eu-NEs

Different formulations of LV-Eu-SNEDDS were prepared by simply mixing the SNEDDS components (i.e., the Eu oil plus Maisine CC mixture, surfactant TPGS, and co-surfactant propylene glycol), and the mixture was vortexed for 5 min. It was left for 12 h to achieve equilibrium in a shaking water bath at 37 °C and 100 rpm [29].

### 4.6. Assessment of the LV-Eu-SNEDDS

#### 4.6.1. Emulsification Ability

The instantaneous emulsification capacity and clarity of the prepared LV-Eu-SNEDDS were used to assess the NE efficiency via visual inspection [30].

#### 4.6.2. Determination of Droplet Size of the Different LV-Eu-SNEDDS

Certain volumes of the formulated nanodispersions were diluted with double distilled water (ratio 1:10 *v*/*v*) to avoid the multiple scattering effect and overcome the effect of sample viscosity. The intended samples were utilized to determine the NEs’ droplet size and compare the sizes of different samples by the dynamic light-scattering technique (Zetatrac, Microtrac, Montgomeryville, PA, USA) [28].

#### 4.6.3. Stability Studies

Formulations that passed the heat-cool test were subjected to various temperature changes to confirm their thermodynamic stability [46]. The LV-Eu-SNEDDS passed through three sequential freeze–thaw cycles (freezing at −25 °C for approximately 12 h and, then, thawing at 25 °C for another 12 h). After these cycles, the final samples were analyzed for droplet size. The samples’ stability indexes were determined by comparing the obtained droplet size with that in initial measurements, using the following equation [31]:

(3) Stability index =([Initial size−Change in size]/Initial size)×100

### 4.7. Optimization of LV-Eu-SNEDDS

The optimization procedure of the LV-Eu-SNEDDS was accomplished as per the aims stated in Table 1. The optimization was performed using the Eu oil/Maisine CC (A), TPGS (B), and propylene glycol (C) in levels with “in range” specifications. The lowest value for the droplet size and the highest value for the stability index were selected for the response variables.

### 4.8. Preparation and Characterization of the Optimized LV-Eu-Nanoemulgel

The gelling agent used to convert the optimized LV-Eu-SNEDDS to nanoemulgels was Carbopol 934. In brief, 25 mL of the optimized LV-Eu-SNEDDS containing 20 mg/mL of LV were diluted with 75 mL of double distilled water; then, 1.5% Carbopol 934 and 0.1% methylparaben were dispersed in the diluted sample and stirred at 250 rpm until the Carbopol 934 was homogeneously dispersed. The dispersion obtained was treated with a desired quantity of triethanolamine to obtain a pH of 5.5. Afterward, the LV-Eu-nanoemulgel was stored in the refrigerator for 24 h before any further characterization to get rid of any entrapped air bubbles. The LV loading within the prepared gels was determined by dissolving 1 g of the prepared gel with 9 mL of methanol and sonicating it for 10 min in a water bath sonicator; the concentration of LV in the resultant dispersion was determined by measuring the UV absorbance at 246 nm. Different LV-loaded nanoemulgels (shown in Table 7) were prepared in the same manner [47].

#### 4.8.1. Rheological Evaluation of the Optimized LV-Eu-Nanoemulgel

The rheological behavior of the optimized LV-Eu-nanoemulgel (formulation A) was compared with that of the plain Carbopol hydrogel (formulation E). One gram of each sample was characterized at 25 ± 1 °C using a Brookfield viscometer and spindle 52. The shear rates of 2, 10, 20, 30, 40, 50, and 60 s^−1^ were employed in the study. The flow curves of the studied samples were constructed, followed by the determination of Farrow’s constant (n) using the following equation [48]:

(4) Log G =n Log F −Log η

where G is the shear rate, η is the viscosity, F is the shear stress, and n is the Farrow’s constant.

#### 4.8.2. In Vitro Release of the Different Formulations of LV-Eu-SNEDDS-Loaded Gels

An in vitro drug release study was done for various preparations, including (1) a hydrogel loaded with the optimized LV-Eu-SNEDDS (A); (2) an aqueous dispersion of an LV-Eu-SNEDDS without the Carbopol 934 gelling agent (D); and (3) an aqueous dispersion of LV 2% (Susp). In this method, a USP dissolution apparatus (type I, basket type; DT 700 LH device, Erweka GmbH DT 700, Heusenstamm, Germany) was used. In the beginning, the prepared formulations were placed in separate cylindrical tubes that had a diameter of 2.7 cm and a length of 6 cm. The release media was stirred at 50 rpm and maintained at 37 ± 0.5 °C. This dissolution test was performed for 6 h. Then, the percentage of LV released at each time interval was determined by measuring the absorbance at 246 nm. All measurements were conducted in triplicate, and data were presented as the mean ± standard deviation (SD).

### 4.9. In Vitro Cell Viability Assay

A previously described method employing 3-[4,5-dimethylthiazol-2-yl]-2,5-diphenyltetrazolium bromide (MTT) was adopted to assess the in vitro cytotoxicity of the prepared formulations against the HSC3 cell line. As depicted in Table 7, the used formulation was the hydrogel loaded with the optimized LV-Eu-SNEDDS (formulation A), which was compared with formulations B, C, D, E, and F. The HSC3 cells were obtained from metastatic lymph node tumors of squamous cell carcinoma in a 60-year-old male patient’s tongue. Fresh explanted tumor tissues were used as a medium for subculturing of the migrating and proliferating epithelial cells [36]. Trypsin-EDTA (0.25%) was used to trypsinize the HSC3 cells at a density of 5 × 104 cells/mL, and then the mixture was added to a 96-well plate (5000 cells/well). One day later, all of the medium was replaced with DMEM (Dulbecco’s modified eagle medium). 5-Fluorouracil was used in the control cells, and the remaining cells were treated with the other tested formulations at a concentration of 10 to 50 μg/mL. After 72 h of incubation, 0.2 mg/mL of MTT in 0.1 mL of DMEM was added to each well plate and incubated for 2 to 3 h. Dimethylsulfoxide (DMSO) replaced the DMEM so that the developed formazan could be dissolved. Then, the absorbance was estimated using a microplate reader (Biotek Synergy, Santa Clara, CA, USA) at 540 nm. Finally, the half-maximal inhibitory (IC_50_) values were determined following the construction of the dose–response curve. The antiproliferative activity of the best-performing formulation was tested against normal HSC3 cells.

### 4.10. Caspase-3 Enzyme Assay

The caspase-3 enzyme assay was carried out for formulations listed in Table 7. At first, cells were cultured in Roswell Park Memorial Institute Medium (RPMI 1640), which contained 10% fetal bovine serum at 37 °C. Cells were further lysed using a cell extraction buffer. The obtained lysate was diluted using a standard buffer as per the assay range for active human caspase-3 traces, and the cells were plated in DMEM (100 μL) at a concentration of 1.3 to 1.9 × 10,000 cells/well. Each sample was inoculated for 24 h in a 96-well plate before the caspase-3 enzyme assay. The assay was performed (as per the kit instructions from USCN Life Science, Wuhan, China) using spectrophotometry at 450 nm [36].

### 4.11. Statistical Analysis

Collected data were analyzed using the one-way analysis of variance (ANOVA) followed by the post-hoc Tukey HSD test for multiple comparisons, and the level of significance was set at a *p*-value of less than 0.05 using SPSS software (version 22, Chicago, IL, USA). The obtained data were tested for normality using the K–S test.

## Figures and Tables

**Table 1:** Projected trial formulation compositions and observed responses of LV-Eu loaded self-nanoemulsion (SNEDDS) formulations, as recommended by the mixture design.

Formulation Code	Independent Variables (Mixture Components)			Ependent Variables		
	A	B	C	Y_1_ (nm)	Y_2_ (%)	PDI
SNEDD-1	0.123	0.427	0.448	147 ± 1.2	86 ± 1.0	0.05 ± 0.002
SNEDD-2	0.120	0.350	0.530	170 ± 0.5	73 ± 0.5	0.30 ± 0.010
SNEDD-3	0.123	0.427	0.448	145 ± 1.1	87 ± 0.2	0.10 ± 0.004
SNEDD-4	0.132	0.500	0.368	122 ± 2.1	93 ± 1.3	0.32 ± 0.005
SNEDD-5	0.198	0.357	0.444	75 ± 0.2	75 ± 0.3	0.09 ± 0.001
SNEDD-6	0.198	0.357	0.444	76 ± 0.5	76 ± 0.5	0.25 ± 0.011
SNEDD-7	0.216	0.433	0.350	80 ± 1.2	89 ± 1.0	0.29 ± 0.004
SNEDD-8	0.184	0.406	0.409	80 ± 1.4	84 ± 1.3	0.17 ± 0.002
SNEDD-9	0.168	0.452	0.378	99 ± 3.1	91 ± 2.2	0.18 ± 0.006
SNEDD-10	0.146	0.376	0.476	102 ± 2.7	80 ± 2.0	0.23 ± 0.003
SNEDD-11	0.132	0.500	0.368	120 ± 1.01	94 ± 3.1	0.28 ± 0.005
SNEDD-12	0.280	0.350	0.370	110 ± 0.9	71 ± 0.9	0.33 ± 0.012
SNEDD-13	0.254	0.395	0.350	96 ± 0.5	82 ± 0.8	0.19 ± 0.008
SNEDD-14	0.235	0.364	0.400	89 ± 1.0	78 ± 1.2	0.22 ± 0.003
SNEDD-15	0.123	0.427	0.448	147 ± 2.1	86 ± 1.5	0.20 ± 0.006
SNEDD-16	0.120	0.350	0.530	170 ± 2.3	73 ± 0.4	0.09 ± 0.008

**Table 2:** Regression analysis results for Y_1_ and Y_2_ responses.

Dependent Variables	R^2^	Adjusted R^2^	Predicted R^2^	F-Value	*p*-Value	Adequate Precision
Y1	0.9987	0.9967	0.8595	494.12	0.0001	69.33
Y2	0.9926	0.9880	0.9761	215.13	0.0001	40.72

**Table 3:** Actual and predicted values of the optimized LV-Eu-SNEDDS.

Solution	Eu/Ma Oil Mixture %	TPGS %	Propylene Glycol %	Droplet Size (nm)	Stability Index (%)	Desirability
Predicated value	0.182	0.468	0.350	83.7	92.7	0.927
Experimental value	0.182	0.468	0.350	85	93	0.927

**Table 4:** Results of the K–S test of normality performed on capsase-3 enzyme data of all formulations.

Formulation	Sample Size	Mean **±** SD	D-Value	*p*-Value
A	6	810 ± 103	0.3603	0.3327
B	6	400 ± 26	0.2834	0.6265
C	6	330 ± 33	0.2222	0.8714
D	6	670± 65	0.2410	0.8049
E	6	270 ± 50	0.2311	0.8612
F	6	55 ± 6.4	0.2144	0.8949

**Table 5:** Tukey HSD test results.

Treatments Pair	Tukey HSD Q Statistic	Tukey HSD *p*-Value	Tukey HSD Inference
A vs. B	17.1126	0.0010053	*p* < 0.01
A vs. C	20.0505	0.0010053	*p* < 0.01
A vs. D	5.5557	0.0056425	*p* < 0.01
A vs. E	22.2993	0.0010053	*p* < 0.01
A vs. F	31.5448	0.0010053	*p* < 0.01
B vs. C	2.9380	0.3254137	insignificant
B vs. D	11.5569	0.0010053	p < 0.01
B vs. E	5.1867	0.0110464	*p* < 0.05
B vs. F	14.4322	0.0010053	*p* < 0.01
C vs. D	14.4949	0.0010053	*p* < 0.01
C vs. E	2.2487	0.5977232	insignificant
C vs. F	11.4942	0.0010053	*p* < 0.01
D vs. E	16.7436	0.0010053	*p* < 0.01
D vs. F	25.9891	0.0010053	*p* < 0.01
E vs. F	9.2455	0.0010053	*p* < 0.01

**Table 6:** Experimental plan of mixture design (component levels and selected responses).

Component	Level		Response	Goal
	Low	High		
Eu/Ma oil mixture level; (A)	0.12	0.28	Mean globule size (Y_1_) Stability Index (Y_2_)	Minimize Maximize
TPGS surfactant level; (B)	0.35	0.5		
Propylene Glycol cosurfactant Level; (C)	0.35	0.53		

**Table 7:** Details of samples prepared for the characterization and evaluation of the LV-Eu-nanoemulgels.

Formulation	Composition
A	Hydrogel loaded with optimized LV-Eu –SNEDDs
B	Hydrogel loaded with NE formulated with castor oil instead of Eu/Ma oil
C	Hydrogel loaded with NE formulated without LV
D	Aqueous dispersion LV-Eu –SNEDDs without using of carbopol 934 gelling agent
E	Physical mixture of LV and Eu oil
F	Carbopol 934 hydrogel (plain)

## Data Availability

All data available are reported in the article.

## Supplementary Materials

The following supporting information can be downloaded at: https://www.mdpi.com/article/10.3390/gels8030176/s1, Table S1: Input data on k = 5 independent treatments; Table S2: Descriptive statistics of your k = 5 independent treatments; Table S3: One-way ANOVA of your k = 5 independent treatments; Table S4: Scheffé results; Table S5: Bonferroni and Holm results: all pairs simultaineously compared; Table S6: Bonferroni and Holm results: only pairs relative to A simultaineously compared.
