# Supplementary material for: Repurposing Lovastatin Cytotoxicity against the Tongue Carcinoma HSC3 Cell Line Using a Eucalyptus Oil-Based Nanoemulgel Carrier
Source: Gels. 2022 Mar 12;8(3):176. doi: 10.3390/gels8030176 (PMC8954000; doi:10.3390/gels8030176)
Supplement: Supplementary file 1 [file gels-08-00176-s001.zip › gels-1623707-Supplementary.pdf]

gels MDPI

Supplementary Materials

Repurposing Lovastatin Cytotoxicity against the Tongue Carcinoma HSC3 Cell Line Using a Eucalyptus Oil-Based Nanoemulgel Carrier

Waleed Y. Rizg [1,2,*], Khaled M. Hosny [1,2], Samar S. Mahmoud [3], Ahmed K. Kammoun [4], Abdulmohsin J. Alamoudi [5], Hossam H. Tayeb [6,7], Haitham A. Bukhary [8], Moutaz Y. Badr [8], Samar S. A. Murshid [9], Eman Alfayez [10], Sarah A. Ali [11], Rayan Y. Mushtaq [12], and Walaa A. Abualsunun [1]

Table S1. Input data on k=5 independent treatments.

Treatment →	A	B	C	D	E
Input Data →	890<br>894<br>863<br>883<br>639<br>694	400<br>376<br>380<br>376<br>433<br>440	320<br>350<br>365<br>368<br>300<br>280	610<br>600<br>635<br>705<br>756<br>759	55<br>46<br>60<br>59<br>48<br>64

One-way ANOVA with post-hoc Tukey HSD Test For Capsase-3enzyme results with Scheffé, Bonferroni and Holm multiple comparison calculation also provided

Table S2. Descriptive statistics of your k=5 independent treatments.

Treatment →	A	B	C	D	E	Pooled Total
observations N	6	6	6	6	6	30
Sum $\sum xi$	4,863.0000	2,405.0000	1,983.0000	4,065.0000	332.0000	13,648.0000
Mean $\bar{x}$	810.5000	400.8333	330.5000	677.5000	55.3333	454.9333
sum of squares $\sum xi^2$	4,005,751.0000	968,241.0000	661,949.0000	2,779,967.0000	18,622.0000	8,434,530.0000
sample variance s^2	12,857.9000	847.3667	1,313.5000	5,185.9000	50.2667	76,744.8230
sample std. dev. s	113.3927	29.1096	36.2422	72.0132	7.0899	277.0286
std. dev. of mean $SE_{\bar{x}}$	46.2924	11.8839	14.7958	29.3993	2.8944	50.5783

Table S3. One-way ANOVA of your k=5 independent treatments.

source	sum of squares SS	degrees of freedom νν	mean square MS	F statistic	*p*-value
treatment	2,124,325.2000	4	531,081.3000	131.0992	2.2204e-16
error	101,274.6667	25	4,050.9867		
total	2,225,599.8667	29			

Table S4. Scheffé results.

treatments pair	Scheffé TT-statistic	Scheffé p-value	Scheffé inferfence
A vs B	11.1484	2.2708 x10^{-9}	p<0.01
A vs C	13.0624	8.0739 x10^{-11}	p<0.01
A vs D	3.6194	0.0273414	p<0.05
A vs E	20.5505	2.7756 x10^{-15}	p<0.01
B vs C	1.9140	0.4701396	insignificant
B vs D	7.5290	3.6144 x10^{-6}	p<0.01
B vs E	9.4022	6.6485 x10^{-8}	p<0.01
C vs D	9.4430	6.1203 x10^{-8}	p<0.01
C vs E	7.4882	3.9594 x10^{-6}	p<0.01
D vs E	16.9312	2.5113 x10^{-13}	p<0.01

Table S5. Bonferroni and Holm results: all pairs simultaineously compared.

treatments pair	Bonferroni and Holm TT-statistic	Bonferroni p-value	Bonferroni inferfence	Holm p-value	Holm inferfence
A vs B	11.1484	3.4219 x10^{-10}	p<0.01	2.3953x10^{-10}	p<0.01
A vs C	13.0624	1.1413 x10^{-11}	p<0.01	9.1305 x10^{-12}	p<0.01
A vs D	3.6194	0.0130732	p<0.05	0.0026146	p<0.01
A vs E	20.5505	0.0000	p<0.01	0.000	p<0.01

B vs C	1.9140	0.6713473	insignificant	0.0671347	insignifi-cant
B vs D	7.5290	6.9749 x10^{-7}	p<0.01	2.7900 x10^{-7}	p<0.01
B vs E	9.4022	1.0960 x10^{-8}	p<0.01	5.4800 x10^{-9}	p<0.01
C vs D	9.4430	1.0063 x10^{-8}	p<0.01	6.0379 x10^{-9}	p<0.01
C vs E	7.4882	7.6751 x10^{-7}	p<0.01	2.3025 x10^{-7}	p<0.01
D vs E	16.9312	3.3307 x10^{-14}	p<0.01	2.9976 x10^{-14}	p<0.01

Table S6. Bonferroni and Holm results: only pairs relative to a simultaineously compared.

**treatments pair**	**Bonferroni and Holm TT-statistic**	**Bonferroni p-value**	**Bonfer-roni infer-fence**	**Holm p-value**	**Holm infer-fence**
A vs B	11.1484	1.3687 x10^{-10}	p<0.01	6.8437 x10^{-10}	p<0.01
A vs C	13.0624	4.5652 x10^{-12}	p<0.01	3.4239 x10^{-12}	p<0.01
A vs D	3.6194	0.0052293	p<0.01	0.0013073	p<0.01
A vs E	20.5505	0.000	p<0.01	0.000	p<0.01
